# Assortative mating among Lake Malawi cichlid fish populations is not simply predictable from male nuptial colour

**DOI:** 10.1186/1471-2148-9-53

**Published:** 2009-03-05

**Authors:** Jonatan Blais, Martin Plenderleith, Ciro Rico, Martin I Taylor, Ole Seehausen, Cock van Oosterhout, George F Turner

**Affiliations:** 1 Département de Biologie, Québec-Océan, Université Laval, Ste-Foy, Québec, GIK 7P4, Canada; 2 Department of Biological Sciences, University of Hull, Hull HU6 7RX, UK; 3 Estación Biológica de Doñana, CSIC, Av M^a ^Luisa s/n Pabellón del Perú, Apartado de correos 1050, 41013, Sevilla, Spain; 4 School of Biological Sciences, Bangor University, Bangor, Gwynedd LL57 2UW UK; 5 Aquatic Ecology and Evolution, Institute of Zoology, University of Bern, Baltzerstr. 6, CH-3012 Bern, Switzerland; 6 EAWAG Swiss Federal Institute for Aquatic Science and Technology, Department of Fish Ecology and Evolution, Center of Ecology, Evolution and Biogeochemistry, CH-6047 Kastanienbaum, Switzerland

## Abstract

**Background:**

Research on the evolution of reproductive isolation in African cichlid fishes has largely focussed on the role of male colours and female mate choice. Here, we tested predictions from the hypothesis that allopatric divergence in male colour is associated with corresponding divergence in preference.

**Methods:**

We studied four populations of the Lake Malawi *Pseudotropheus zebra *complex. We predicted that more distantly-related populations that independently evolved similar colours would interbreed freely while more closely-related populations with different colours mate assortatively. We used microsatellite genotypes or mesh false-floors to assign paternity. Fisher's exact tests as well as Binomial and Wilcoxon tests were used to detect if mating departed from random expectations.

**Results:**

Surprisingly, laboratory mate choice experiments revealed significant assortative mating not only between population pairs with differently coloured males, but between population pairs with similarly-coloured males too. This suggested that assortative mating could be based on non-visual cues, so we further examined the sensory basis of assortative mating between two populations with different male colour. Conducting trials under monochromatic (orange) light, intended to mask the distinctive male dorsal fin hues (blue v orange) of these populations, did not significantly affect the assortative mating by female *P. emmiltos *observed under control conditions. By contrast, assortative mating broke down when direct contact between female and male was prevented.

**Conclusion:**

We suggest that non-visual cues, such as olfactory signals, may play an important role in mate choice and behavioural isolation in these and perhaps other African cichlid fish. Future speciation models aimed at explaining African cichlid radiations may therefore consider incorporating such mating cues in mate choice scenarios.

## Background

One of the most significant recent development in speciation theory has been the increased attention given to sexual selection as an evolutionary force capable of rapidly inducing reproductive isolation among populations [1]. Sexual selection is thought to have played an important role in major adaptive radiations, such as those of Hawaiian *Drosophila *[2,3] and East African cichlid fishes [4-6]. Because many closely related species differ in sexually dimorphic male breeding colour, many studies on cichlid fishes from Lakes Malawi and Victoria have emphasized the possible role of female choice of male nuptial colour as a driving force for speciation [5,7-10]. The observations that individual females can have preferences for different male colour patterns and that these preferences lead to reproductive isolation between incipient species have been used to model speciation in cichlid radiations [e.g. [11-14]]. The breakdown of assortative mating between a pair of sympatric Lake Victoria cichlid species in turbid waters [15], the breakdown of assortative mating during laboratory experiments under monochromatic light [9], and the observation that non-hybrid females prefer hybrid males that have the colours of conspecifics [16] provided evidence that female mating preferences for male courtship hue are important in reproductive isolation in Lake Victoria cichlids.

Cichlid fishes specialised to live on rocky shores are known to be philopatric and poor dispersers, with significant genetic structure among populations isolated by habitat discontinuities [17,18]. It has been proposed that differentiation of mating traits under divergent sexual selection among allopatric populations may contribute to the high diversity of these lineages [4-6,19]. Allopatric colour variants are common among the rock-dwelling haplochromine species of Lake Malawi [20], and a substantial proportion of the estimated species richness of these fish is based on the allocation of species status to allopatric colour variants [21,22]. Laboratory mate choice trials with closely related populations have indicated substantial, albeit incomplete, assortative mating between populations that differ substantially in male colour and random mating between populations with more similar colour [6], suggesting that colour differentiation may play a pivotal role in prezygotic isolation.

Similar adult male coloration can be found in populations from widely separated localities within Lake Malawi [23]. Molecular phylogenetic analysis using microsatellites [24] and genome-wide surveys of Amplified Fragment Length Polymorphisms (AFLPs) [25] indicate that some of these populations have evolved similar colours independently. A powerful way of testing the role of male colour-female preference coevolution in producing prezygotic isolation among these species is to test whether assortative mating of populations can be predicted based on similarity of independently derived colour patterns. Indeed, if male colour tightly coevolved with female preference, we would expect to find cases of parallel speciation whereby unrelated populations that independently evolved similar male coloration would freely interbreed. We would furthermore predict that females discriminate against males from more closely-related populations with different colour patterns [6,23,25]. That parallel speciation may occur in nature has been shown for sympatric benthic and limnetic populations of sticklebacks (*Gasterosteus *spp.) in North American lakes that have adapted to different ecological conditions [26,27]. Although sympatric species of these fish are largely reproductively isolated by mate choice, allopatric species of similar ecotype that adapted independently to similar conditions in different lakes (e.g. benthics from different lakes) lack behavioural isolation. These findings raise the possibility that under similar ecological conditions, populations in different places can evolve in parallel and upon secondary contact form one polyphyletic species [28].

However, recent laboratory studies [29] have shown that the strong tendency of females of the Lake Malawi cichlid *Pseudotropheus emmiltos *to choose conspecific males broke down when only visual cues were available, but was restored when the experimental design allowed olfactory cues while still preventing direct physical contact. Males of the sympatric species used had distinct dorsal fin markings and background hue that would have been visible under the experimental conditions. This suggests that male colour and female preference for male colour may not have coevolved closely with each other in this species.

On this background, we examine the hypothesis that parallel evolution of colour in Lake Malawi cichlids resulted in parallel speciation. We took advantage of naturally occurring colour diversity by using four allopatric populations of the rock-dwelling *Pseudotropheus zebra *species complex from Lake Malawi. Territorial males of all populations are blue with black vertical bars, but males of two populations have orange dorsal fins, while those from the other two sites have blue dorsal fins. According to Schluter and Nagel [27], at least three criteria must be met to demonstrate parallel speciation. First, populations exposed to the same conditions must be phylogenetically independent so that shared traits responsible for reproductive isolation evolved separately. Second, populations exposed to different environmental conditions must be reproductively isolated. Third, populations exposed to the same environmental conditions must not be reproductively isolated. Here, we selected a pair of orange/blue populations from the northwestern part of the lake, *P. emmiltos *and *P. zebra*, and another orange/blue pair from the southeastern area, *P. thapsinogen *and *P. zebra*. The northwestern populations are closely related and belong to a clade of northwestern and central eastern populations according to AFLP data [25]. The southeastern orange population (*P. thapsiongen*) was shown to have evolved independently from the northwestern orange *P. emmiltos *population and to be more closely related to the southeastern blue *P. zebra *population than from the northwestern populations according to microsatellite analysis [25]. These populations therefore satisfy the first criterion of parallel speciation established by Schluter and Nagel [27].

We tested whether partial assortative mating among allopatric populations is the result of co-evolution between female preference and male colour, and investigated the roles of different sensory modalities in female mate choice experiments following two different approaches. First, we specifically address Schluter and Nagel's [27] second and third criteria. Under the scenario of parallel speciation through colour-preference co-evolution, we predict random mating between distantly-related populations with similar male colours and assortative mating between more closely-related populations with clearly different male colours. Second, in order to further evaluate the role of colour divergence as the trait hypothesized to cause reproductive isolation in the system, we artificially manipulated colour and other cues in mate choice trials involving one of these population pairs (*P. emmiltos *and northern *P. zebra *from Nkhata Bay). We tested the importance of visual cues by comparing mate choice under normal (white) light and monochromatic light [9], while other cues were manipulated by comparing female choice of males under white light behind transparent partitions [29].

## Methods

### a) Experimental animals

**Figure 1:**
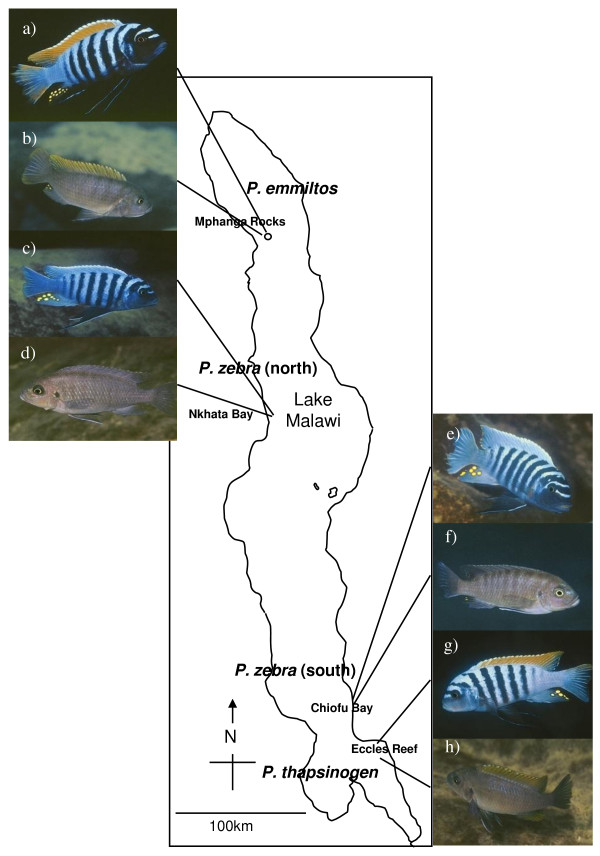
**Map of Lake Malawi showing sampling sites of study populations**. Male (a) and female (b) *Pseudotropheus emmiltos *(northern orange) from Mphanga Rocks; male (c) and female (d) *Pseudotropheus zebra *(northern blue) from Nkhata Bay; male (e) and female (f) *Pseudotropheus zebra *(southern blue) fromChiofu Bay and male (g) and female (h) *Pseudotropheus thapsinogen *(southern orange) from Eccles Reef.

The study populations were members of the *Pseudotropheus zebra *species complex, cichlid fish endemic to Lake Malawi. The taxonomy of this group is confused, and these fish are sometimes assigned to the genus or subgenus *Maylandia *or the genus *Metriaclima*. Many allopatric colour variants have been assigned species names, while others have not. Territorial males of all four study populations are pale blue with strongly contrasting black vertical bars, but populations differ in the colour of the dorsal fin. Male *P. zebra *from Nkhata Bay (11°36'S, 34°18'E) on the northwestern shore and Chiofu Bay (13°13'S, 34°52'E) on the southeastern shore have blue dorsal fins (henceforth referred to as 'northern blue' and 'southern blue', respectively). Male *P. thapsinogen *from Chimwalani (Eccles) Reef (13°46'S, 34°58'E) off the southeastern shore and male *P. emmiltos *from Mpanga Rocks (10° 25'S 34°16'E) near the northwestern shore have orange dorsal fins and are referred to as 'southern orange' and 'northern orange', respectively (Figure 1). *P. thapsinogen *(southern orange) males have yellow throat membranes, whereas those of the northern orange males (*P. emmiltos*) are dark grey. These throat membranes are normally not visible unless protruded beneath the gill-cover as part of a lateral display. However, these displays are typically part of antagonistic rather than courtship interactions, and therefore it is unlikely that females use this trait in mate choice. The taxonomy, morphology and ecology of these populations were discussed by Stauffer et al. [30] and the biology of these and related 'mbuna' species reviewed by Genner & Turner [31]. Fish used were wild-caught, although some first-generation laboratory stock bred from wild-caught parents were used in experiment 2. Housing and maintenance conditions have been described previously [6,29].

### b) Experimental procedures

#### Experiment 1: Test of parallel speciation through colour-preference coevolution

Levels of behavioural reproductive isolation were measured in glass aquaria 6 m long × 0.7 m wide × 0.4 m deep under a 'partial partition' design [6,32], where males were confined to territories of floor dimensions 60 × 70 cm using plastic grids adjusted to allow the smaller females to pass through and spawn with any preferred male. Standard fluorescent lamps were used throughout the study. Although *Pseudotropheus *species express a UV-sensitive cone opsin, examinations of photographs of these and related species taken under UV light did not reveal any UV-specific markings, simply a greater degree of luminosity of the bright blue parts of their colour, which does not vary noticeably between the populations. It is therefore unlikely that different results could have been obtained with UV-enhanced tubes. Terracotta flower pots were provided as spawning caves. Four males (size-matched within 5 g) and 10–15 females of each of two populations were present throughout the experiment. Males' positions in the tank were randomized, except that two males of the same population were not allowed to be neighbours. After at least one clutch had been produced by females of both populations, one or two males of each population were removed, and replaced by size-matched males of the same population, and all males were moved to new territories. For each population comparison, 5–7 rearrangements were carried out, and 8–11 males per population used to minimise the influence of individual variation in male attractiveness. Courtship behaviour is known to be highly conserved in haplochromine cichlids across broad taxonomic scales [33] and we did not observe any differences in courtship behaviour among populations. It was estimated that 63% of males used sired part or all of one or more clutches. Four population combinations were tested. Of these, two reciprocal trials compared more distantly-related populations with similar male colours: 1) *P. emmiltos *females choosing between males of the same population and *P. thapsinogen *males and vice versa (northern v southern orange) and 2) *P. zebra *females from Nkhata Bay choosing between males of the same population and *P. zebra *males from Chiofu Bay and vice versa (northern v southern blue). The other two reciprocal trials compared more closely-related and geographically proximal populations with distinct male colours: 1) *P. emmiltos *females choosing between males of the same population and *P. zebra *males from Nkhata Bay and vice versa (northern orange v blue), and 2) *P. thapsinogen *females choosing between males of the same population and *P. zebra *males from Chiofu Bay and vice versa (southern orange v blue).

Before introduction into the experimental aquarium, all males and females were PIT-tagged for individual identification, and fin-clipped for DNA testing. A small piece of tissue was cut from the soft-rayed part of the dorsal fin and placed in 100% ethanol. Mouthbrooding females were removed from the aquarium *ca*. 5–10 days after spawning and their offspring removed by gently opening the female's mouth and allowing the fry to drop into a tray of water containing an anaesthetic overdose (MS-222). Fry were then preserved in 100% ethanol. Paternity was assigned by genotyping all mothers, potential fathers and 3–15 offspring per clutch at between two and four microsatellite loci: UNH130 [34], UNH002 [35], UME002 [36], and Pzeb2 [37]. DNA was extracted from tissue samples using the HOTSHOT method [38] and amplified using a FAM/HEX/TET-labelled forward primer. PCR conditions consisted of 30 cycles of 30 sec at 95°, 60 sec at 55°, 60 sec at 72° in a 20 μl reaction volume containing 0.25 mM of each dNTP, 2 μl of 10× reaction buffer (Bioline, London, UK) 1.5 mM MgCl_2_, 0.5 U of taq DNA polymerase (Bioline, London, UK), 25 ρmol of each primer and approximately 1 μg of template genomic DNA in a TGradient thermocycler (Biometra, Göttingen, Germany). PCR products were resolved on an ABI 310 (Applied Biosystems, Foster City, U.S.A.), with ROX-labelled internal size standards. Alleles were sized using Genemapper (Applied Biosystems).

#### Experiment 2: Test for role of colour in behavioural isolation

**Figure 2:**
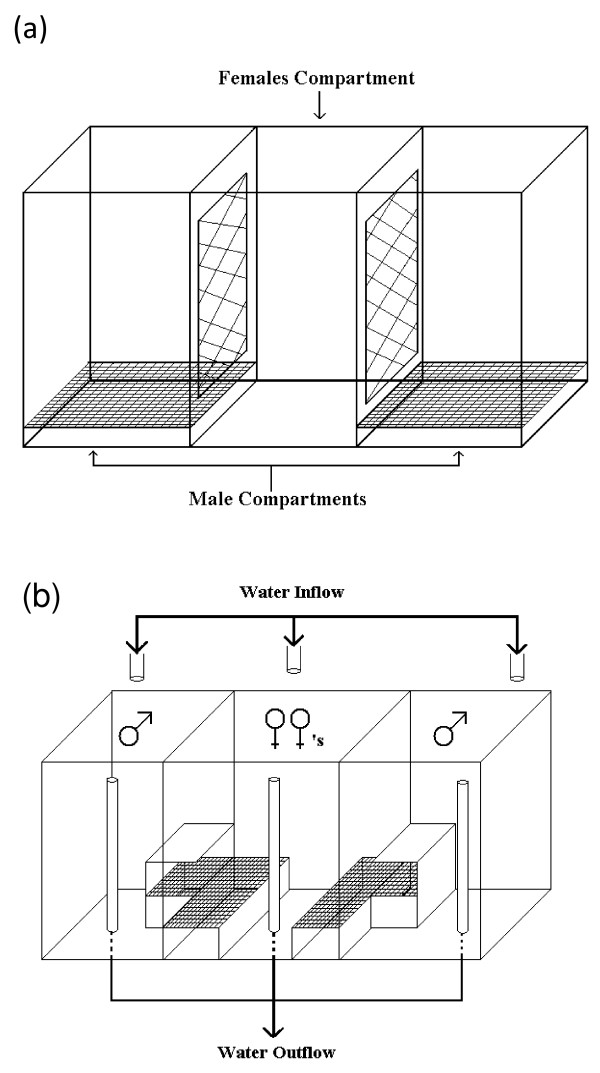
**Experimental set up for experiment 2**. Control and monochromatic light experiments (a), and visual-only experiment (b). Males in the two end compartments courted females from the central compartment.

Experiments were run in aquaria of dimensions 180 × 45 × 33 cm or 200 × 50 × 31 cm in which mate choice was determined from a group of 10–20 females of a single species at a time (northern orange *P. emmiltos *or northern blue *P. zebra *from Nkhata Bay). In all trials, the males were size-matched for weight (± 5 g) and standard length (± 5 mm). Female choice was assayed by counting eggs that had collected underneath mesh false-floors, where they were inaccessible to the females that would normally collect them in their mouths [29]. The male that received the largest number of the eggs was taken to be preferred. Twelve males from each population (northern orange *P. emmiltos*/northern blue *P. zebra *from Nkhata Bay) were used in each experimental treatment. Mate choice was tested under three treatments: (i) *White light with full contact*, in which males were kept in compartments with false floors, separated from a female-only compartment by plastic grids that allowed females to pass through and spawn inside the male compartment, the tank being illuminated with standard fluorescent lights (figure 2a); (ii) *Monochromatic light with full contact*, in which the apparatus was the same as the previous treatment, but the experiment was conducted in a darkroom in which the aquarium lights were covered with a red optical filter (primary red, Lee Filters 106, ). This effectively masked the colour difference between males of the two populations by transmitting light from only the orange and red portion of the white spectrum (500–700 nm), thereby eliminating the difference between the orange and blue dorsal fin colour; (iii) *White light, visual communication only*, in which the tank, illuminated by standard fluorescent lights, was separated into three sections by transparent 5 mm thick acrylic partitions, with the females confined in a central section which had a tray covered with plastic mesh adjacent to each male compartment, which contained an artificial cave used as a spawning site next to the female compartment (figure 2b).

### c) Data analysis

Simulation of parentage assignment of 10,000 randomly generated offspring genotypes in Cervus v. 3.0.3 [39] showed that typing between two to four (average of three) of the four loci used resulted in an 82% successful assignment rate to one of the eight potential fathers given known mother genotype when using a delta LOD criterion with 95% confidence interval and allowing for 1% of mistyped loci. We first typed two loci for all individuals, and progressively increased to three and then four loci whenever unambiguous paternal assignment to one of the eight possible males could not be obtained. At the end of this process, we were able to unambiguously assign paternity to a single male for virtually all fry of all clutches. In most cases, clutches were sired by a single male or by several males of the same population. We found only a few cases of multiple paternity involving males of different populations. Multiple paternity was resolved by a majority consensus rule where the sire of the majority of the first five eggs sampled at random was considered as the preferred male, thus providing an unbiased statistical sample at the population level. To minimise the impact of variation in clutch sizes between broods, final analyses were based on the first 5 eggs typed, or the total, if smaller.

The significance of assortative mating of population pairs was assessed by one-tailed Fisher Exact Tests, which tests the hypothesis of assortative mating at the level of the tested species pair as a whole (at the level of the whole 2 × 2 table). It is thus more powerful than testing individual reciprocal crosses. We also report, for individual reciprocal crossings, one-tailed Binomial Tests on numbers of broods and Wilcoxon tests on the number of eggs typed for each clutch assigned to sires of the two populations. The binomial test is based on the binomial distribution, while the Wilcoxon signed-rank test does not assume anything about the shape of the distribution. The full data set of experiment 1 included broods where analysis of maternal genotypes suggested that they were produced by females that had already produced one brood earlier in the experiment, or broods where males had sired the majority of eggs sampled from a number of broods produced by different females [see Additional file 1]. To ensure that these non-independent points did not impact our conclusions, we analysed a reduced dataset, keeping only the first brood produced by each female and eliminating second or subsequent broods with the same majority sire. This eliminated possible influences of over-representation of particular individual female preferences, particularly successful males, or mate choice copying, although mate choice copying by Malawi cichlids has never been reported, and many of the clutches were spawned weeks or months apart, often after a male had been moved to a new territory.

## Results

### Experiment 1: Test of parallel speciation through colour-preference co-evolution

Eighty-nine clutches were produced and 3–15 fry (mean ± S.E. = 6.6 ± 1.9) from each of these tested for paternity. Of these, 12 (13% of total) were sired by males of different populations. Forty-nine broods with dam or majority sire involved in earlier broods were removed from the original dataset before analysis. Once these broods were eliminated from the data set, only seven clutches sired by males of different populations remained.

#### Test of random mating among similarly coloured populations

Contrary to the prediction of random mating among geographically distant populations of the same colour, Fisher's exact tests indicated significant assortative mating among both pairs of distantly-related populations with similar-coloured males (Table 1). In two populations (southern blue *P. zebra *and northern orange *P. emmiltos*), Binomial and Wilcoxon signed-rank test indicated that males from the female's population were significantly more likely to sire the majority of the clutches (Table 1). Northern blue *P. zebra *and southern orange *P. thapsinogen *females also spawned more often with males from their own population, although not significantly.

**Table 1:** Mate choice trials among populations of *Pseudotropheus zebra *and related species in large arena tanks.

Dam Population	Majority Sire Population		Binomial Test one-tailed *P*	Wilcoxon Test one-tailed *P*	Fisher's Exact Test one-tailed *P*
	NB	SB			
NB	4	0	0.063	*Z *= 1.826, *P *= 0.068	
SB	0	5	**0.031**	*Z *= 2.023, *P *= **0.043**	
					** < 0.01**
					
	NO	SO			
NO	6	0	**0.016**	*Z *= 2.201, *P *= **0.028**	
SO	2	5	0.227	*Z *= 0.507, *P *= 0.612	
					**0.016**
					
	SB	SO			
SB	2	2	0.500	*Z *= 0.000, *P *= 1.000	
SO	0	4	0.063	*Z *= 1.826, *P *= 0.068	
					0.214
					
	NB	NO			
NB	4	0	0.063	*Z *= 1.618, *P *= 0.106	
NO	2	5	0.227	*Z *= 0.676, *P *= 0.499	
					**0.045**

Forty-nine broods from dams or sires involved in previous spawnings were eliminated from the original dataset to ensure data independence. The results were inconsistent with the predictions of the hypothesis of parallel speciation by divergence of male colour and associated female preference, with significant assortative mating between geographically and phylogenetically more distant populations with similarly-coloured males [northern orange *P. emmiltos *(NO) v southern orange *P. thapsinogen *(SO); northern blue *P. zebra *from Nkhata Bay (NB) v southern blue *P. zebra *from Chiofu Bay (SB)] but not between more closely related and geographically proximal populations with differently-coloured males (SO v SB; NO v NB). Shown are the frequencies with which males were estimated to have sired the majority of the offspring genotyped in a clutch. The Binomial Test and Fisher Exact Tests were based on these figures, while the Wilcoxon Test was based on the actual numbers of eggs typed for each clutch assigned to sires of the two populations. Significant p-values (α = 0.05) are in bold.

#### Test of assortative mating among differently-coloured populations

When comparing geographically proximate populations with different colours, Fisher's exact test indicated significant assortative mating only between *P. emmiltos *and *P. zebra *from Nkhata Bay (Table 1). Although female *P. emmiltos*, *P. zebra *from Nkhata Bay and *P. thapsinogen *spawned more often with males of their own populations, assortative mating was not significant in any of the individual reciprocal crosses (Binomial and Wilcoxon tests p > 0.05; Table 1). Females of the southern *P. zebra *population of Chiofu Bay did not differ from random expectation and spawned half of their clutches with *P. thapsinogen *males (Table 1).

### Experiment 2: Test for role of colour in behavioural isolation

Mate choice of female P. emmiltos (northern orange): Under control conditions of full sensory contact under white light, female P. emmiltos spawned exclusively with males of their own (northern orange) population, rather than northern blue P. zebra males (Table 2; Binomial p < 0.0001). This preference persisted in monochromatic light, as females spawned with same-population males in 11 out of 12 trials (Binomial p < 0.005), which does not differ significantly from the control (Fisher's exact test, p ~1). When free contact was blocked, but colour differences were visible under white light, they showed no significant preference, spawning with the same-population male only in 5 of the 12 trials (Binomial p = 0.386). Female choice with visual contact only differed significantly from that in the experiment with full sensory communication under white light (Fisher's exact test, p = 0.04).

**Table 2:** Mate choice trials under treatments to control sensory cues in northern blue *P. zebra *from Nkhata Bay and northern orange *P. emmiltos*

Female	Treatment	Male		Cumulative Binomial Probability (one-tailed)
		*P. emmiltos*	*P. zebra*	
*P. emmiltos* (NO)	Control: free contact; white light	12	0	** < 0.001**
	Treatment 1: free contact, monochromatic light	11	1	**0.005**
	Treatment 2: visual only, white light	7	5	0.386
*P. zebra* (NB)	Control: free contact, white light	5	7	0.386
	Treatment 1: free contact, monochromatic light	7	5	0.386

Female preference for conspecific *P. emmiltos *males was maintained under monochromatic light, but lost when non-visual cues were blocked (response variable is the number of replicates in which the majority of the clutch was laid next to a male of a particular species). Significant p-values (α = 0.05) are in bold.

Mate choice of female P. zebra (northern blue): With full contact under white light, northern blue females' preference for males of their own population was not significant (Table 2; Binomial p = 0.386), spawning with the same-population male in 7 out of 12 trials. Under monochromatic light, same-population males were chosen in 5 out of the 12 trials (Binomial p = 0.386). As northern blue females had not shown significant assortative mating in the control treatment of experiment 2, they were not tested with free contact blocked.

## Discussion

The suggestion that sexual selection may have played a major role in the diversification of the cichlid fishes of the African Great Lakes has been largely inspired by the enormous diversity in colour pattern among the Malawian and Victorian haplochromine cichlids. One of the main assumptions of speciation models by sexual selection so far was that evolutionary divergence in female colour preference was driving the emergence of behavioural isolation [40]. The hypothesis of parallel speciation of populations having independently evolved the same colour patterns examined in this paper derives from this idea. The importance of male colour hue for reproductive isolation has been shown in a pair of Lake Victoria cichlid species where assortative mating broke down under monochromatic light [9] and where non-hybrid females choosing among hybrid males prefer those that have the colour of conspecific males [10]. These species are fully sympatric, differ in visual pigment sensitivities [41] and in their behavioural response to blue and red light [42]. They appear to hybridise more often in habitats with low water transparency [15]. Strong assortative mating based on visual cues (possibly melanin pattern rather than hue) have also been shown in the polymorphic Lake Victoria cichlid *Neochromis omnicaeruleus *[43,44] and the polymorphic Lake Malawi cichlid *Pseudotropheus "*zebra gold" [45].

Between-population assortative mating based on colour has yet to be convincingly demonstrated with Lake Malawi cichlid species. Experiments with some Lake Malawi species have shown that assortative mating can persist when transparent partitions prevented direct contact but not visual communication [40,46,47]. These results suggest a significant role for visual cues in mating preference. However, these cues are not necessarily confined to the male's hue, but could also include male melanin pattern, eggspot size and number, body shape, and male responsiveness to visual stimuli from the female. Indeed, the pair examined by Kidd et al. [40] differ in melanin pattern rather than hue, and Jordan et al. [47] found that assortative mating was maintained even under monochromatic light masking the species' hue differences, implying that some visual features other than hue were used by females. Even when it has been demonstrated that a single cue may be sufficient for assortative mating, other cues may also be influential. For example, olfactory cues may be important in the later stages of courtship, while females are initially attracted by visual signals. If a pair of species had attained reproductive isolation based on the preference for divergent signals in one signal modality, there could subsequently be strong selection to recognise and avoid courting with heterospecifics using other sensory modalities for signalling. In such cases, visual signals could be sufficient to lead to assortative mating in experiments where access to olfactory signals was prevented, even if divergence in olfactory signals had been the initial cause of reproductive isolation or vice versa.

Here, laboratory mate choice tests with allopatric populations of *Pseudotropheus zebra *and related nominal species were inconsistent with the predictions we derived from the hypothesis that parallel correlated divergence in male colour and female preferences had resulted in parallel speciation among these fishes. Firstly, we demonstrated that allopatric populations differing in dorsal fin colour did not show complete assortative mating, and probably should not be considered as biological species. Unexpectedly, allopatric populations with similar colours showed relatively high levels of (partial) assortative mating. This suggests that colour differentiation is not necessarily a prerequisite for the evolution of significant prezygotic isolation.

We predicted that geographically proximal and phylogenetically related populations showing clear differences in male breeding colour would mate assortatively. This prediction was supported in one of the two population pairs with different male colours. By contrast, the second prediction that geographically and phylogenetically distant populations with similar male colour should mate randomly was not verified. Instead, we found assortative mating in both pairs of similarly coloured populations from distant locations. Of course, it is still possible that further comparisons may show evidence for parallel speciation, perhaps a weaker form of incipient parallel speciation, where pairs of independently derived similar-coloured populations may show lower levels of assortative mating than among pairs of more closely-related differently-coloured populations.

Like most studies of behavioural evolution, we are required to make inferences about past events based on knowledge of current populations and their past relationships, and this is often aided by having a robust phylogeny of the study taxa and an estimate of divergence times. Recently, it has been shown that Lake Malawi suffered a major lake level fall of around 580 m until about 70,000 years ago, with present lake levels only being restored within the past 50,000 years [48,49]. At such low lake levels, the nearest refuge for the southern populations used in the present study would have been around Nkhata Bay- the site of one of the northern populations. Given that the northern and southern populations do not yet seem to mate completely assortatively and probably occurred sympatrically 70,000 years ago, it is likely the study populations are descended from a single common ancestor which was split into multiple populations as new habitat patches became available (and were lost or fused) during the rapid expansion of the lake around 60–70,000 years ago. There is no evidence that any of these study populations have ever co-existed in sympatry and maintained complete reproductive isolation.

The two population pairs used in this study were carefully chosen for the similarity of their colour pattern apart from the colour of the dorsal fin (and the yellow throat membrane of *P. thapsinogen*). We were unable to find any other differences among populations in hue or melanin pattern. However, subtle differences cannot be entirely ruled out, although we consider it unlikely.

Another potential limitation concerns the possible influence of colour differences only visible under ultraviolet (U.V.) light. Although we could not find any such differences when examining photographs of the specimens under U.V. light and we found significant assortative mating in many populations without enhanced U.V. lighting, further tests might reveal an impact of U.V. illumination in mate choice in these populations.

Experimentally controlling the effect of visual and non-visual cues revealed that the strong tendency of northern orange (*P. emmiltos*) females to mate with same-population males over northern blue males (*P. zebra*) persisted when male colour differences were masked under monochromatic light. However, assortative mating broke down when direct contact was prevented, even when male colour differences and other visual features should have been apparent under white light. These results are inconsistent with colour being a necessary requirement for the assortative mating observed between northern orange females and northern blue males, and with visual cues being sufficient for reproductive isolation of this pair. They are, however, in agreement with Plenderleith et al.'s [29] results demonstrating the role of olfactory cues for mate choice when *P. emmiltos *females were presented with conspecific males and those of the sympatric *P. fainzilberi*. This is also consistent with the findings of experiment 1 where assortative mating was observed in pairs where colour differences were absent or minimal. The lack of significant assortative mating of *P. zebra *females in experiment 2 compared with experiment 1 is possibly simply due to limited statistical power, but perhaps it may also be because experiment 2 did not allow for mate choice among males of the same population, as only one male of each population was used at a time. Thus, assortative mating might have been reduced when an otherwise attractive male of another population might be chosen in preference to a male of the female's own population that was considered less attractive, perhaps in traits that did not differ among populations.

Future studies might aim at testing the generality of the importance of non-visual cues in other haplochromine species. Among the cues cichlids may use in mate choice, those involving olfactory, acoustic and other non-visual modalities remain little investigated. Plenderleith et al. [29] proposed that olfactory signals may be particularly promising for explaining some cases of assortative mating. Recently, Blais et al. [50] showed that there was evidence of adaptive divergence at MHC class II genes between *P. emmiltos *(northern orange) and the sympatric *P. fainzilberi *and suggested a mechanism by which this divergence may have contributed to reproductive isolation of the pair. MHC genes are known to influence mating behaviour and kin recognition in many fishes through olfaction [e.g. [51-53]] and to lead to female rejection of mates with highly dissimilar MHC multilocus genotypes in humans, Malagasy giant jumping rats, *Hypogeomys antimena*, and threespine sticklebacks [52,54,55]. There is therefore one potential candidate gene for odour-based mate choice known to have diverged between a pair of the *P. zebra *complex.

Haplochromine cichlids are usually strongly sexually dimorphic in colour. We do not yet know whether olfactory signals involved in mate choice are expressed by both sexes. If so, it would greatly increase the likelihood that mate preferences may be learned by imprinting on the mother during mouthbrooding, a situation that may enhance the probability of speciation [56]. Sexual imprinting on maternal traits, perhaps olfactory signals, has recently been shown in a pair of Lake Victoria cichlid fish [57]. By contrast, assortative mating based on male breeding dress is less likely to be aided by imprinting, as male haplochromine cichlids play no role in parental care and male nuptial colours are not or only weakly expressed in females [57]. Moreover, because of their chemical nature, olfactory signals might be more likely than others to vary with small differences in ecological traits such as diet, microhabitat or parasite infections [50,58,59], perhaps augmenting the likelihood of reproductive isolation among populations.

## Conclusion

The three principal implications of the findings presented here are that: (i) behavioural mating preferences in cichlid fish depend, in some populations, not on colour, but on other forms of sensory communication, which in some cases is not visual; (ii) between-population divergence in male colour does not necessarily imply divergent female preferences, so parallel evolution of male colour is not necessarily coupled with parallel evolution of female mating preference; (iii) sensory cues involved in behavioural isolation among haplochromine cichlids may vary among species and lakes and so speciation models based on one particular mate choice scenario may not apply to whole or all radiations of cichlids. This last point is worth bearing in mind because the type of trait involved in species isolation might provide important clues about which evolutionary force has driven population divergence and speciation.

## Authors' contributions

JB performed microsatellite genotyping, statistical analyses, and drafted the manuscript. MP performed behavioural experiments. CR provided critical review and discussion of draft versions. MIT performed microsatellite genotyping and contributed to preparation of the final manuscript. OS provided critical review and discussion of draft versions and supervised the data collection for experiment 1. CVO contributed with critical discussion of draft versions. GFT coordinated the study and was involved throughout the design, analysis, interpretation and writing of the work. All authors have read and approved the final manuscript.

## Supplementary Material

Additional file 1 **Results of mate choice trials involving *Pseudotropheus zebra *and related species populations.** The data presented are statistical analyses of the full data set, including female multiple spawnings and male multiple sirings, in mate choice trials involving either geographically and phylogenetically distant or proximal *Pseudotropheus zebra *and related species populations. Click here for file
