# Supplementary material for: Assortative mating among Lake Malawi cichlid fish populations is not simply predictable from male nuptial colour
Source: BMC Evol Biol. 2009 Mar 5;9:53. doi: 10.1186/1471-2148-9-53 (PMC2667177; doi:10.1186/1471-2148-9-53)
Supplement: Additional file 1 — Results of mate choice trials involving Pseudotropheus zebra and related species populations. The data presented are statistical analyses of the full data set, including female multiple spawnings and male multiple sirings, in mate choice trials involving either geographically and phylogenetically distant or proximal Pseudotropheus zebra and related species populations. [file 1471-2148-9-53-S1.doc]

**Mate choice trials between geographically and phylogenetically distant populations of *Pseudotropheus zebra* and related species with similarly-coloured males in large arena tanks.**

|  | Majority Sire  Population | | Binomial Test  one-tailed *P* | Wilcoxon  one-tailed *P* | Fisher’s Exact Test one-tailed *P* |
| --- | --- | --- | --- | --- | --- |
| Dam Population |  |  |  |  |  |
|  | NB | SB |  |  |  |
| NB | 6 | 2 | 0.145 | *Z* = 0.791, *P* = 0.215 |  |
| SB | 0 | 18 | **< 0.001** | *Z* = 3.69, *P* ***<* 0.001** |  |
|  |  |  |  |  | **<0.001** |
|  | NO | SO |  |  |  |
| NO | 10 | 0 | **< 0.001** | *Z* = 2.78, *P* **< 0.01** |  |
| SO | 3 | 12 | **0.018** | *Z* = 2.03, *P* = **0.021** |  |
|  |  |  |  |  | **< 0.001** |

Some broods are from the same dams or sires and therefore the data are not all independent. Results were inconsistent with predictions of the hypothesis of parallel speciation by divergence of male colour and associated female preference, with significant assortative mating between both geographically and phylogenetically more distant population pairs with similarly-coloured males [northern orange *P. emmiltos* (NO) v southern orange *P. thapsinogen* (SO); northern blue *P. zebra* from Nkhata Bay (NB) v southern blue *P. zebra* from Chiofu Bay (SB)]. In three of four crossing directions, males from the same population were more likely to sire the majority of the clutches. In the fourth case, NB females also spawned more often with their own males, although not significantly so. Shown are the frequencies with which males were estimated to have sired the majority of the offspring genotyped in a clutch. The Binomial and Fisher Exact Tests were based on these figures, while the Wilcoxon Test was based on the actual numbers of eggs typed for each clutch assigned to sires of the two populations. Significant p-values (α = 0.05) are in bold.

**Mate choice trials between geographically and phylogenetically related populations of *Pseudotropheus zebra* and related species with differently-coloured males in large arena tanks.**

|  | Majority Sire Population | | Binomial Test one-tailed *P* | Wilcoxon Test one-tailed *P* | Fisher’s Exact Test one-tailed *P* |
| --- | --- | --- | --- | --- | --- |
| Dam Population |  |  |  |  |  |
|  | SB | SO |  |  |  |
| SB | 5 | 7 | 0.774 | *Z*= 0.485, *P* = 0.628 |  |
| SO | 0 | 8 | **< 0.01** | *Z*= 2.563, *P* = **0.001** |  |
|  |  |  |  |  | 0.051 |
|  | NB | NO |  |  |  |
| NB | 7 | 0 | **< 0.01** | *Z*= 2.322, *P* = **0.01** |  |
| NO | 2 | 9 | **0.033** | *Z*= 1.54, *P* = 0.062 |  |
|  |  |  |  |  | **< 0.01** |

Some broods are from the same dams or sires and therefore the data are not all independent. The results supported the predictions of the hypothesis of parallel speciation by divergence of male colour and associated female preference in the northern pair, with significant assortative mating between northern orange *P. emmiltos* (NO) v northern blue *P. zebra* from Nkhata Bay (NB). There was also a marginally non-significant (p = 0.051) signal for southern orange *P.* *thapsinogen* (SO) v southern blue *P. zebra* from Chiofu Bay (SB). There was evidence that males from the same population were more likely to sire the majority of the clutches in three of four crossing directions. In the fourth case, SB females did not show any tendency to assortative mating, actually spawning more often with heterospecific SO males. Shown are the frequencies with which males were estimated to have sired the majority of the offspring genotyped in a clutch. The Binomial and Fisher Exact Tests were based on these figures, while the Wilcoxon Test was based on the actual numbers of eggs typed for each clutch assigned to sires of the two populations. Significant p-values (α = 0.05) are in bold.
